# Monitoring Changes in Depression Severity Using Wearable and Mobile Sensors

**DOI:** 10.3389/fpsyt.2020.584711

**Published:** 2020-12-18

**Authors:** Paola Pedrelli, Szymon Fedor, Asma Ghandeharioun, Esther Howe, Dawn F. Ionescu, Darian Bhathena, Lauren B. Fisher, Cristina Cusin, Maren Nyer, Albert Yeung, Lisa Sangermano, David Mischoulon, Johnathan E. Alpert, Rosalind W. Picard

**Affiliations:** ^1^The Depression Clinical and Research Program, Massachusetts General Hospital, Boston, MA, United States; ^2^The Media Lab, Massachusetts Institute of Technology, Cambridge, MA, United States; ^3^Department of Psychology, University of California, Berkeley, Berkeley, CA, United States; ^4^Janssen Research and Development, San Diego, CA, United States; ^5^Department of Psychiatry and Behavioral Sciences, Montefiore Medical Center and Albert Einstein College of Medicine, Bronx, NY, United States

**Keywords:** artificial intelligence, sensors, digital phenotyping, assessment, depression

## Abstract

**Background:** While preliminary evidence suggests that sensors may be employed to detect presence of low mood it is still unclear whether they can be leveraged for measuring depression symptom severity. This study evaluates the feasibility and performance of assessing depressive symptom severity by using behavioral and physiological features obtained from wristband and smartphone sensors.

**Method:** Participants were thirty-one individuals with Major Depressive Disorder (MDD). The protocol included 8 weeks of behavioral and physiological monitoring through smartphone and wristband sensors and six in-person clinical interviews during which depression was assessed with the 17-item Hamilton Depression Rating Scale (HDRS-17).

**Results:** Participants wore the right and left wrist sensors 92 and 94% of the time respectively. Three machine-learning models estimating depressive symptom severity were developed–one combining features from smartphone and wearable sensors, one including only features from the smartphones, and one including features from wrist sensors–and evaluated in two different scenarios. Correlations between the models' estimate of HDRS scores and clinician-rated HDRS ranged from moderate to high (0.46 [CI: 0.42, 0.74] to 0.7 [CI: 0.66, 0.74]) and had moderate accuracy with Mean Absolute Error ranging between 3.88 ± 0.18 and 4.74 ± 1.24. The time-split scenario of the model including only features from the smartphones performed the best. The ten most predictive features in the model combining physiological and mobile features were related to mobile phone engagement, activity level, skin conductance, and heart rate variability.

**Conclusion:** Monitoring of MDD patients through smartphones and wrist sensors following a clinician-rated HDRS assessment is feasible and may provide an estimate of changes in depressive symptom severity. Future studies should further examine the best features to estimate depressive symptoms and strategies to further enhance accuracy.

## Introduction

Depression is among the most common and disabling mental health disorders, with a worldwide prevalence of more than 300 million people (1). Despite the availability of many evidence-based treatments for Major Depressive Disorder (MDD), ~50% of US individuals with depression are not treated (2) and only 26% of those with past year MDD receive adequate treatment (3). Barriers to treatment include stigma, limited access to specialty care, poor symptom monitoring, and limited patient insight into symptoms (4). Due to the low availability of specialized care for depression, the disorder is often identified and managed in primary care settings (5, 6). However, the time constraints of primary care physicians (PCPs) make thorough symptom monitoring difficult, which may in turn contribute to inadequate or delayed treatment (7). In the absence of resources for close symptom monitoring, many PCPs follow the American Psychiatric Association's (APA) Practice Guideline for the Treatment of Patients with MDD and wait up to 12 weeks before adjusting medications in the absence of a response (8). Delaying time to medication change may prolong suffering, reduce the likelihood of complete remission (9), and increase risk for relapse (10). Further, a high percentage of patients who experience full remission, will experience a relapse (10, 11), the treatment for which is frequently delayed due to providers' expectation that remitted patients will contact them when deteriorating. Methods enabling passive, real-time symptom monitoring may facilitate early detection of response or non-response to treatment, or of depression relapse and allow expedited delivery of relief to patients.

Over the past decade, the development of wristband and smartphone-embedded sensors has facilitated the objective measurement of numerous hallmark symptoms of depression and the passive monitoring of behavioral indices of low mood (12). Consistent with the well-established association between low levels of socialization and depressive symptoms (13), recent work shows that severe depressive symptoms are associated with shorter duration of incoming and outgoing calls, and fewer incoming and outgoing phone calls and daily text messages (13–18). Anhedonia and low energy level can manifest as reduced physical activity (19, 20), which can be measured using GPS and motion sensors embedded in smartphones or wearable sensors. It has also been documented that more severe depressive symptoms and worse mood levels are negatively correlated with a higher amount of time the phone screen is on, a higher number of entertainment applications (apps) used, and an overall time of interaction with the smartphone (18, 21). Finally, dysregulated sleep, a common problem in depression, can be measured relatively well by wearable activity sensors (22).

Preliminary studies suggest that aggregates of smartphone-based passive features are useful in predicting daily mood (23) and presence of depressive symptoms (24). While findings in the field of sensor-based assessment in psychiatry are promising, critical gaps are still present. Most studies evaluating passive monitoring have examined depressive symptoms among patients with bipolar disorders. Those studies conducted with patients with depression have mostly relied on self-report questionnaires to assign diagnoses and/or measure variation in symptoms and train the models. This has resulted in possible reliability problems, since these investigations have primarily focused on detecting presence or absence of depression rather than on assessing its severity, and they have shown overall low accuracy (14–18, 23–27). Moreover, despite evidence showing the existence of physiological indices that are markers of depressed mood (28), that can be continuously collected through wearables (29), and which can be combined with smartphone data to assess mood (27), only two studies have combined these data streams in models to monitor changing depressive symptoms (26, 27). However, both of these studies included previously described limitations such as reliance on self-report questionnaires to train their models and low accuracy. Only one relied on exclusive passive data collection (27).

The current study aimed to address these gaps by evaluating the feasibility and performance of using a machine-learning model that combines physiological features passively recorded by wearable sensors and smartphone features to assess depressive symptoms severity among patients diagnosed with Major Depressive Disorder. Models estimating depressive symptom severity only from smartphone features and only from wristband-based features, were also evaluated. Finally, we examined which features from the smartphone and wearable sensors were most informative in estimating depressive symptom severity. Based on previous reports, we hypothesized a strong correlation between estimates of depressive symptom severity from the model combining smartphone and wearable features and clinician-rated depressive symptom scores.

## Materials and Methods

### Participants

Participants were recruited using standard methods (e.g., flyers). Forty-one participants with MDD were enrolled and 31 completed the study. Seven participants dropped out after the screening visit, two after visit three, and one after visit two. Participants were between the ages of 19 and 73 (*M* = 33.7, SD = 14), and primarily female = 23 (74%). Ethnic distribution was White = 22 (71%), Hispanic/Latino = 4 (23%), Asian = 5 (16%), Haitian/Black/African-American = 4 (12%), American Indian/Alaskan = 1 (3%), mixed-race = 2 (6%), and other = 1 (3%). At screening, participants on average had severe depressive symptoms [28-item Hamilton Depression Rating Scale (30) = 22.74; SD = 7.38].

### Procedure

The study encompassed six in-person visits, daily smartphone-delivered surveys, and passive assessment over 9 weeks. The first screening visit included an informed consent procedure, a blood test to assess for potential medical contributors to depressed mood, and clinician-rated symptom assessment. During the second visit (baseline visit) the monitoring app was downloaded onto participants' phones, wristband sensors were applied, and in-person clinical assessments and self-report measures were completed. The remaining four clinical visits occurred bi-weekly over 8 weeks.

Inclusion criteria were current MDD (per the DSM-IV) (31), Hamilton Depression Rating Scale (HDRS-28) (30) score of > 18 at screening, measurable electrodermal activity, use of an Android smartphone as a primary device, ownership of a computer or tablet compatible with the wristband sensors, and daily internet access. Exclusionary criteria were drug or alcohol use disorder within the past 3 months, lifetime history of psychosis, mania, hypomania, epilepsy, or seizure disorder, current untreated hypothyroidism, unstable medical condition or cognitive impairment, acute suicide or homicide risk, current electroconvulsive therapy treatment, vagal nerve stimulation therapy, deep brain stimulation, transcranial magnetic stimulation therapy, or phototherapy, concurrent participation in other research studies involving investigational agents or blinded randomization to treatment, and inability to comprehend and communicate in English.

The protocol was approved by the Institutional Review Boards of Massachusetts General Hospital and Massachusetts Institute of Technology.

### Measures

#### Clinician-Administered

Mini International Neuropsychiatric Interview (MINI): The MINI, a structured diagnostic interview for major psychiatric disorders, was administered during the screening visit to determine current MDD and rule out exclusionary diagnoses (32).

Hamilton Depression Rating Scale (HDRS): The HDRS-28 is a 28-item clinician-rated assessment scale to assess depressive symptoms (30). The HDRS was administered six times: during the screening visit, 1 week later during the baseline visit, and every other week from visit third to sixth. The HDRS-17 (33), one of the methods most commonly employed to measures change in depressive symptoms severity in treatment for depression clinical trials, was derived from the HDRS-28, and used as dependent variable. The HDRS-28 was administered by clinical staff at the Depression Clinical Research Program (DCRP). Staff at the DCRP has been extensively trained in the use of the HDRS by using videotapes and live interviews of patients. Recent assessment of inter-rater reliability between DCRP clinicians in diagnosing MDD and measuring severity of depression has yielded kappas > 0.75, indicating satisfactory agreement (based on internal data).

#### Sensors

Participants were instructed to wear two E4 Empatica (34) wristbands, one on each wrist, for 22 h a day/7 day a week (with 1 h/day for charging and 1 h/day to upload data). Participants could upload the data at any point of the day. The E4 measures electrodermal activity (EDA), peripheral skin temperature, heart rate (HR), motion from the 3-axis accelerometer and sleep characteristics using actigraphy.

#### Smartphone Sensor Data

Mobile-based social interactions (e.g., number of calls, texts), activity patterns (e.g., still, walking), and number of apps used were tracked through the phone app MovisensXS (35) that was downloaded onto participants' phones. For privacy reasons, no phone usage details were recorded, including content of calls/texts, app names, and internet use content. MovisensXS has been successfully used to securely and confidentially collect behavioral and self-reported mood data (36).

### Data Analyses

Data analyses included evaluation of the acceptability of the E4 devices and of the performance of a model using features from smartphones and wristband sensors to estimate depressive symptoms severity assessed with the HDRS. Acceptability was evaluated by examining adherence of wearing the E4. We processed adherence for the entire study, and also after excluding the days when the data were missing because of technical problems including E4 sensors malfunctioning, problems with uploading the data to the server, or problems with the network connection. While most technical problems were promptly addressed, some resulted in the loss of data from multiple consecutive days because it took time to substitute the faulty sensors or get support from the technology providers.

**Figure 1:**
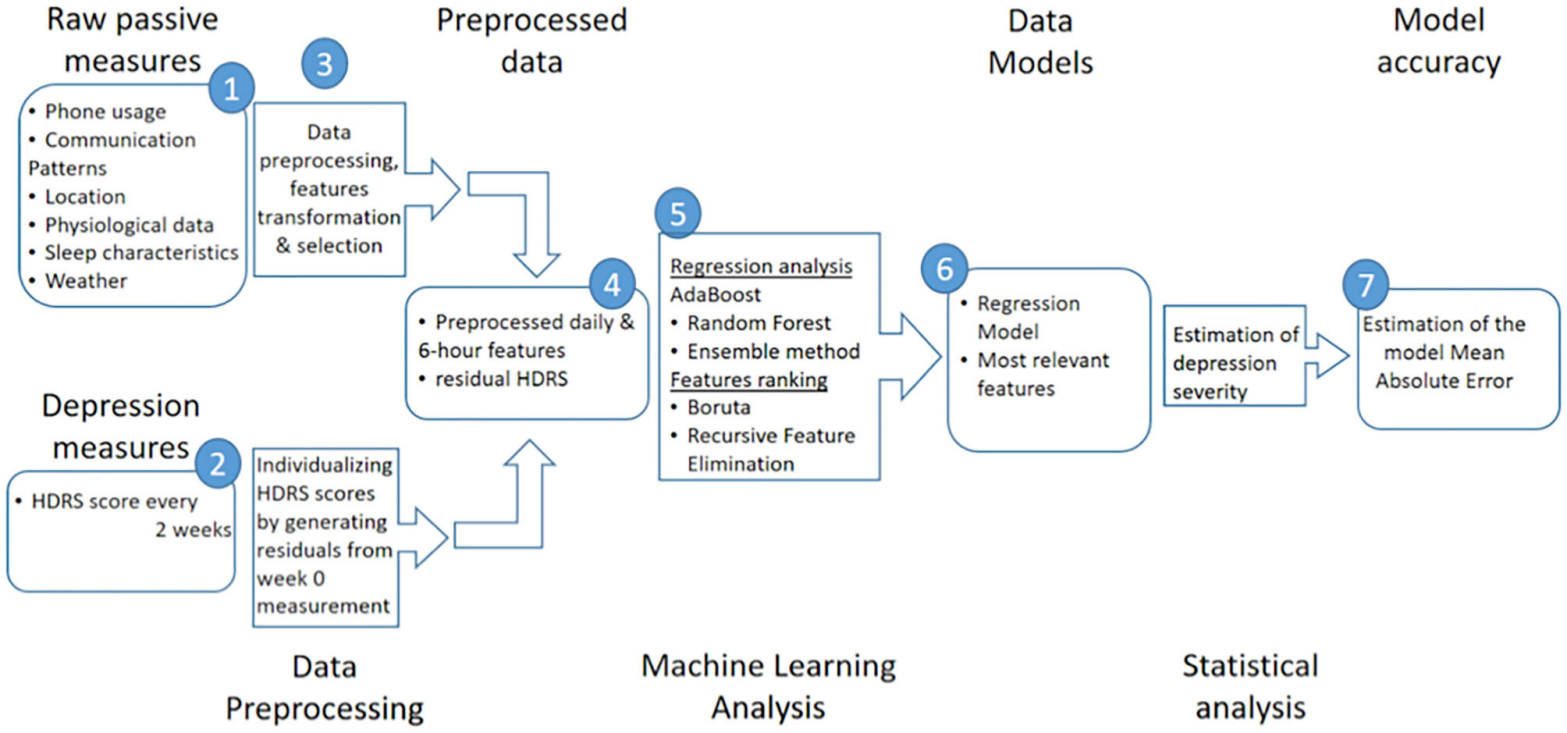
Machine learning process steps.

The model was developed through several steps as shown in Figure 1. All features were preprocessed, transformed and calculated for four 6-h intervals and for daily aggregates (over the course of 24-h). We built upon and expanded our previous work (37) and encoded a comprehensive list of physiological and behavioral features including EDA, motion, sleep, phone usage, call and messaging behavior, app usage, and location change patterns (see comprehensive list in Supplementary Table 1). Preprocessing of EDA, motion, and sleep features was improved relative to our previous work by adding EDA features calculated during the time when there is no motion (identified by the accelerometer sensor) and by adding normalized EDA features (see additional information in Section A1 in Supplementary Material). Moreover, a location preprocessing step was included by down-sampling location data-points to one recording per 5 min followed by extrapolating missing location latitude and longitude values. Consistent with other investigators (18), more semantic features based on location: time spent at home, transition time, total distance traveled, and weighted stationary latitude and longitude standard deviation (A.3. in Supplementary Material) were added. Given that mood can be influenced by the weather (38, 39), location was used to retrieve historical weather data from the DarkSky API (40) and features related to temperature, pressure, humidity, sunrise and sunset time, cloud cover and wind were included. The final dataset included 877 features (Supplementary Table 1), of which 404 derived from the wearables and 473 from the mobile sensors. Similar to our previous work (37), we addressed the potential problem of overfitting by reducing the dimensionality of features using linear and non-linear transformations (see more details in A.2. in Supplementary Material). The resulting 25 transformed features are difficult to interpret as they are non-linearly derived from the original features. Hence, we used the Boruta algorithm described below to identify the most informative features to estimate HDRS scores.

#### Personalization

The HDRS scores included in the training and test model were the residual values obtained subtracting HDRS score of the screening visit from the HDRS scores collected during later visits (visits 2, 3, 4, 5, and 6). Residualized HDRS scores allowed to account for the heterogeneous presentation of depressive symptoms.

#### Multimodal Model Training and Estimation of Depressive Symptom Severity

**Figure 2:**
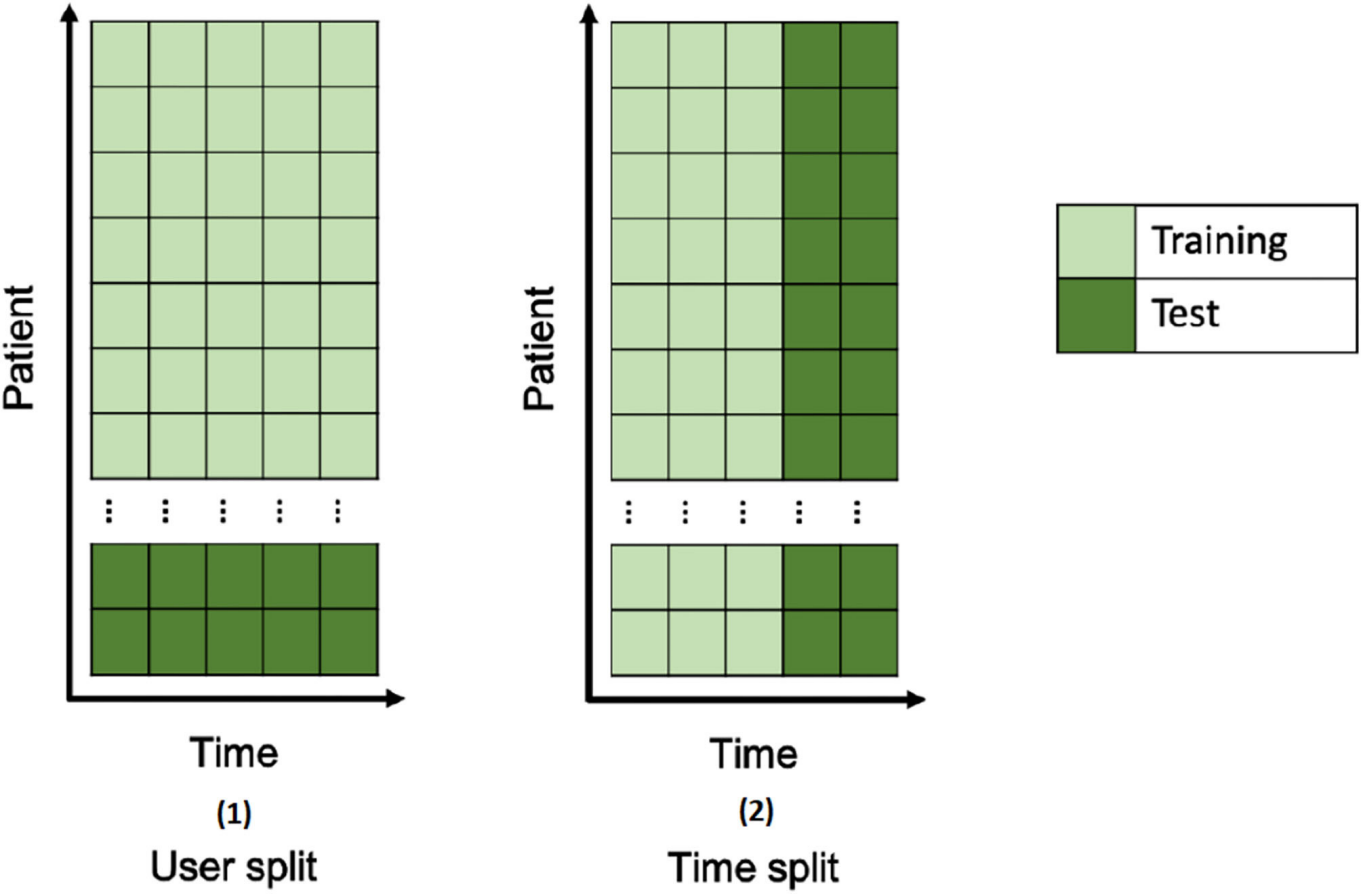
Two evaluation scenarios for splitting the data into training and test sets.

The model to estimate residualized HDRS scores was built by using features from the wrist and smartphone sensors from the same day (midnight-to-midnight) of the HDRS administration. Machine learning techniques used to build the model were average ensemble of boosting (41) and random forest (42). To avoid overfitting, multiple dimensionality reduction and feature transformation techniques were applied to the raw features and we performed 10-fold-cross-validation (41) during training (see more details in A.2. in Supplementary Material); After the parameters of the model were learnt, the model was tested on a separate hold-out test set of data. The model was evaluated under two deployment scenarios resembling different clinical settings (Figure 2). Specifically, the data were split into training and test sets differently and, subsequently each resulting model had slightly different parameters: (1) In the *user-split scenario*, a set of 20% of participants were randomly selected as a hold-out test set and the remaining 80% of participants provided a training set. With this evaluation methodology, the performance of the model was assessed as if it were trained on specific clinic patients or a population, and then used to estimate depressive symptom variation among other patients. (2) In the *time-split scenario*, the first three HDRS scores following the screening visit from all participants were pooled along with the first HDRS score to form the training set and the remaining two HDRS scores were pooled to form the hold-out test set. With this evaluation methodology, the performance of the model was assessed as if it were trained on three visits from one patient, and then used to estimate future depressive symptom variations for the same patient.

In both scenarios HDRS scores were residualized on the HDRS from the screening visit. The performance of the model on the hold-out test set in each scenario was expressed as the mean absolute error (MAE), or the average absolute difference between the clinician-based HDRS and the model-estimated HDRS and as the root mean square error (RMSE). Per standard procedure (42), model performances in each scenario were compared to the MAE and RMSE of estimates based on: (1) group median HDRS values, (2) individual HDRS values at the screening visit, and (3) individual median value of the HDRS from the three visits following the screening visits (this was possible only in the time split scenario). Despite the simplicity of these estimates, most previous work has not outperformed individual baselines in similar settings (42). Correlations were also conducted between the clinician-based HDRS and the HDRS estimated in the testing set of the two scenarios (A.3. in Supplementary Material). Two additional models were developed following the procedure described above, one including only features from the wearables and one including only features from the smartphones, and their performance were compared with the model combining all the features.

#### Features Ranking

To identify the most important and non-redundant features for the estimation of the HDRS scores from passive data, the 877 features included in the regression models were examined using the Boruta algorithm (43) which uses the wrapper method around the Random Forest algorithm.

## Results

### Acceptability

On average, participants uploaded about 17 and 15.5 h of daily data from left and right-hand sensors respectively. This corresponds to 77 and 70% adherence considering that participants were asked to use 2-h each day to charge the E4 and to upload data, which led to a maximum of 22 h of data per day. The average adherence increased to 94% and 92% from the left and right hand respectively, after we excluded the days with technical problems (Supplementary Figures 1, 2).

### Performance

**Figure 3:**
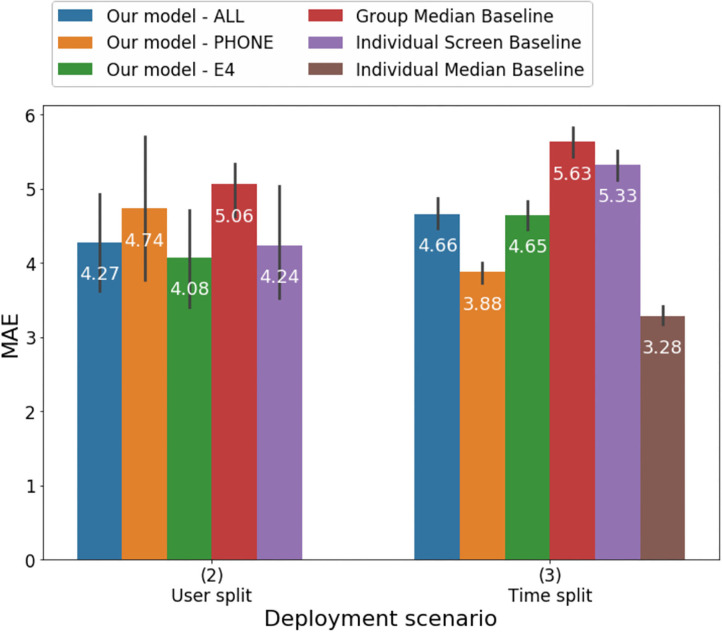
Mean absolute error of predicting HDRS using different models under the User split and Time split scenarios.

**Figure 4:**
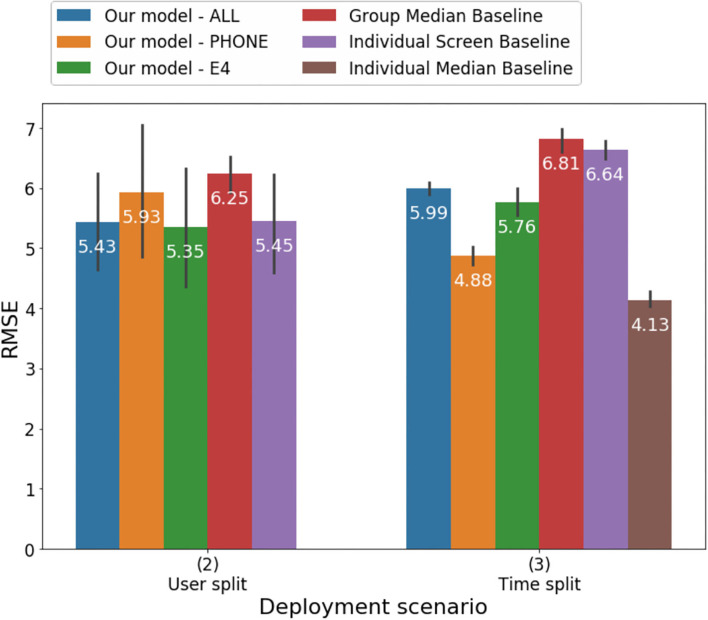
Root mean square error of predicting HDRS using different models under the User-split and Time-split scenarios.

Results of the analyses estimating HDRS score from models including passive features as well as baseline models are illustrated in Table 1 and Figures 3, 4. Overall, all of the machine learning models performed similarly with MAE ranging between 3.88 and 4.74 and correlations between the clinician-rated HDRS scores and the estimated HDRS scores ranging between 0.46 and 0.7 (Table 1). Of the three machine-learning models tested in the time-split scenario, the lowest mean absolute error (MAE) was obtained by the model that included only features from the mobile [*F* (2,12) = 19.04, *p* < 0.002]. When the three models were tested with the user-split scenario, they all performed about the same [*F* (2,12) = 0.55, *p* < 0.59] with the lowest MAE obtained by the model using only the features from the wearable. Thus, it is not possible to speculate as to whether one modality outperformed the others. The machine learning models provided more accurate estimates than those based on group median and individual screen models but not better than those based on individual median in the time split scenario. However, these differences were also not significant. Thus, the normalized MAE ranged between 7.5 and 9.1%, as the HDRS-17 ranges from 0 to 52. Using the Boruta algorithm (43) 39 features were defined as important for the estimation of the HDRS scores, one feature remained undecided, and the remaining features were identified as irrelevant to the outcome variable. The features that were retained were in the following categories: mobile phone engagement, activity level recorded by the mobile sensors, and skin conductance and HRV features from the wearables (Table 2). Notably, 54% of the 39 informative features that were retained by the Boruta analyses were from the mobile phone and 46% of all the informative features were from the wearables.

**Table 1:** Performance of all the models estimating HDRS under the User-split and Time scenarios.

	**User split**			**Time split**		
	**RMSE (SD)**	**MAE (SD)**	**r (95% CI)**	**RMSE (SD)**	**MAE (SD)**	**r (95% CI)**
All features Mobile + Wearable	5.43 (1.03)	4.27 (0.87)	0.57 (0.42, 0.72)	5.99 (0.14)	4.66 (0.25)	0.5 (0.45, 0.55)
Mobile	5.93 (1.45)	4.74 (1.24)	0.46 (0.18-0.74)	4.88 (0.19)	3.88 (0.18)	0.7 (0.66, 0.74)
Wearable	5.35 (1.16)	4.08 (0.9)	0.56 (0.39, 0.73)	5.76 (0.3)	4.65 (0.24)	0.54 (0.49, 0.59)
Group median baseline	6.24 (0.39)	5.06 (0.47)	NA	6.82 (0.23)	5.63 (0.24)	NA
Individual screen baseline	5.45 (1.1)	4.24 (0.99)	0.65 (0.5, 0.8)	6.64 (0.19)	5.33 (0.23)	0.42 (0.38, 0.46)
Individual median baseline	NA	NA	NA	4.13 (0.17)	3.28 (0.16)	0.81 (0.80, 0.82)

**Table 2:** Illustration of the features selected by the Boruta algorithm ranked by importance.

1. Average time phone screen was on over 24 h	2. Average skin conductance response difference between right and left wrist recorded during motionless intervals over 24 h	3. Average SD of the location latitude and longitude from 12 p.m. to 6 p.m. (*location_totalStd_12 to 18*)
4. Average duration phone screen was on from 8 a.m. to 6 p.m.	5. Average location latitude over 24 h	6. Average skin conductance level mean difference between right and left wrist during motionless intervals over 24 h
7. Longitude standard deviation from 6 a.m. to 12 p.m.	8. Average location latitude from 8 a.m. to 6 p.m.	9. HRV root mean square of successive differences between normal heartbeats on the right wrist between 6 p.m. and 12 a.m.
10. Median latitude between 12 p.m. and 6 p.m.	11. Median time phone screen was on over 24 h	12. Average of skin conductance response amplitude peaks on the left wrist processed during motionless intervals over 24 h
13. HRV average of the SD of N-N intervals from 12 a.m. to 6 p.m. on the right wrist	14. Standard deviation of the Location latitude between 6 a.m. and 12 p.m.	15. Average location latitude from midnight to 6 a.m.
16. Average duration phone screen was on from 12 p.m. to 6 p.m.	17. SD of the Location latitude between 12 p.m. and 6 p.m.	18. SD of durations when phone screen was on from 12 p.m. to 6 p.m.
19. SD of the Location latitude between 8 a.m. and 6 p.m.	20. Average HRV power of the low frequency signal band over 24 h measured on right wrist	21. HRV average of the SD of N-N intervals over 24 h on the left wrist
22. HRV average of SD of N-N intervals from 6 p.m. to 12 a.m. on the right wrist	23. SD of HRV power of the low frequency signal band over 24 h measured on right wrist	24. SD of location latitude over 24 h
25. Average skin conductance level on the left wrist during motionless intervals over 24 h	26. SD of durations when phone screen was on from 8 a.m. to 6 p.m.	27. HRV Average of the SD of N-N intervals from 6 p.m. to 12 p.m. on the left wrist
28. HRV average of the SD of N-N intervals over 24 h on the right wrist	29. Skin conductance response difference right and left wrist processed during motionless intervals over 24 h	30. Number of times the phone screen was on from 12 p.m. to 6 p.m.
31. Average SD of the location latitude and longitude from 6 a.m. to 12 p.m.	32. Root mean square of successive differences between normal heartbeats over 24 h on the right wrist	33. Total time the phone screen was on from 12 p.m. to 6 p.m.
34. Average latitude 12 p.m. to 6 p.m.	35. Skin conductance response difference right and left wrist processed during motionless intervals over 24 h	36. Average power of the high frequency band of the HRV signal over 24 h on the right wrist
37. SD duration phone was on over 24 h	38. SD of the IBIs for all sinus beast from 6 p.m. to 12 p.m. on the right wrist	39. Root mean square of successive differences between normal heartbeats from 6 p.m. to 12 a.m. on the right wrist

*SD, standard deviation; HRV, Heart Rate Variability*.

## Discussion

The study examined the feasibility and performance of a model measuring changes in depressive symptoms severity that combined behavioral and physiological indices of depression collected passively by smartphone and wrist sensors. Adherence was decreased by technological problems, which accounted for 17 and 22% decrease in adherence on the left-hand and right-hand wristbands respectively, a finding that also suggested that fixing the reliability of network access, connectivity, and sensor, laptop, and phone hardware would lead to more than 90% adherence.

Our study was the first to evaluate behavioral and physiological features, collected entirely passively among a sample of carefully characterized adult individuals with MDD. Previous evaluations of models to estimate depression passively have primarily relied on examining correlations between estimated and observed symptoms (18, 26, 27). However, indices of associations do not allow a granular evaluation of the accuracy of the models and of the magnitude of the difference between estimated and actual values, impacting scalability. The current study evaluated the performances of the models estimating the severity of the symptoms by using multiple indices including MAE, RMSE and correlations. Correlations between predicted and observed severity of depressive symptoms ranged from moderate to strong (r ranging between 0.46 and 0.7). The correlation between observed and estimated depression in the time-split model including features from the mobile phone (*r* = 0.7) was the strongest and was higher than the one of a previous model combining features from the fitbit and from smartphones (the best model yielded an r^2^ = 0.44 or *r* = 0.66) (27) and the one of a model aggregating mobile-based and physiological features (*r* = 0.58) (26). Notably, despite the high magnitude of the correlations MAE ranged between 3.8 and 4.74 which may be too high of an inaccuracy for the model to be scalable.

Similarly, even though the model with mobile features in the time-split scenario performed significantly better than the others, it is unclear whether a test of significance is the most appropriate metric to compare these models and to determine whether a model is meaningfully better. In the future, criteria should be identified to evaluate when models to estimate depression severity may be deemed adoptable in clinical setting. Our models' RMSE values ranged between 4.88 and 5.99 and were higher than a model estimating self-report depressive symptoms among adolescents that included number of steps, activity level, number of SMS, and calls yielded (RMSE = 2.77) (44). The difference in results may be due to the fact that the studies employed different measures of depression. Our study, together with previous findings, suggests that including different features in the models may have significant implications on accuracy.

In order to develop a thorough method for passive assessment of depressive symptoms the study evaluated a model including behavioral as well as physiological features, which have both been described as important markers of depression. Given the high number of features that could be collected by mobile and wearable sensors the study examined which of the features included in the machine learning model were the most important, and not redundant, to estimate depressive symptoms severity. Out of the 877 features that were initially included in the model, 39 were retained that were primarily related to activity level, mobile phone engagement, skin conductance, and HRV. Our finding that activity level was among the most important features of our model estimating depressive symptoms severity is consistent with previous reports (23, 24). Saeb et al. (18) first showed that mobility pattern, recorded by phone sensors, is associated with depression, O'Brien et al. (45) documented that physical activity was low among individuals with late life depression related to healthy controls and Cao et al. (44) documented that activity level was associated with low mood among adolescents. Similarly, our finding suggesting that engagement with the phone is an important predictor of depression severity is consistent with previous studies (18).

As anticipated, physiological features were important predictors of depressive symptoms. Previously, it had been shown that wearables-based recording of skin conductance could be leveraged to detect high stress and to distinguish between high and low mental health groups of individuals (25). Our findings expand on previous reports by showing that not only commonly considered physiological features play a role in estimating depressive symptoms severity but that also features related to asymmetry of right and left skin conductance response may be important. Previously we have posited that right and left asymmetry may be a better indication of arousal than one-sided EDA measurements (46). Our finding of the role of HRV in predicting depressive symptoms is consistent and extends previous reports. Previous models estimating mood states with moderate accuracy have included measures of heart rate (HR) (27, 44) and HRV (26). Moreover, Cao et al. (44) reported that in a model including HR features recorded by the fitbit, light exposure, and sleep one of the most important features to estimate mood variation among 18 individuals with MDD was HR. Contrary to what was anticipated, we did not find sleep being a critical feature in our model. This finding may have been due to the fact that sleep features in our model were derived from up to 48 h prior to the HDRS assessments and features capturing a longer time period may be needed to characterize depressive symptoms.

Thus, our findings suggested that behavioral as well as physiological features contributed to our model's accuracy. However, while the ubiquity of smartphones makes their use in monitoring symptoms highly scalable, passive collection of physiological indices may be less feasible due to the high cost of wearables. Given the number of behavioral and physiological features that can be collected passively, further studies are critical to examine which features, or aggregate of features, are the most critical to identify models which are the most parsimonious, feasible, and scalable.

Findings should be interpreted in the context of several limitations. Sample size was small and participants experienced low variability of depressive symptoms during the course of the study (e.g., average standard-deviation of within-user HDRS scores was 3.7 and, on average, the HDRS score from baseline to the last assessment decreased by 15%); it is unclear whether the model will have the same performance among patients with higher variability of depressive symptoms. Future studies may examine larger samples and evaluate whether other machine learning models such as Deep Neural Networks may improve performance. It is important to highlight that the Boruta method evaluates the importance of the features in the context of all the features in the model. Thus, a model including a combination of features different from ours may yield different results.

In sum, our findings highlight that machine learning may be a feasible method to estimate HDRS scores using passive monitoring based on mobile and physiological features. However, while evidence is accumulating that these models may have moderate accuracy, larger studies are needed to further evaluate them. Moreover, it is still unclear which features may be critical to develop the best models. Significant advances in the development of sensors and methodologies to analyze big data have created a new frontier of digital phenotyping, defined as the “moment-by-moment quantification of the individual-level human phenotype *in-situ* using data from smartphone and other personal devices (12).” To date, digital phenotyping has included the collection of behavioral data such as mobility patterns (via accelerometer) and socialization (via call and texts) (12). Evidence is accumulating suggesting that physiological sensing may also be included in the effort to objectively characterize changes in depression severity. Digital phenotyping of depression can be leveraged as a clinical tool and may allow a more seamless continuous treatment. In the future, sensor-based systems could privately and continuously track the symptoms of consenting patients and share this information with providers. Rather than relying on patients to reach out in times of need, providers could use these data to offer expeditious and personalized support when symptoms worsen. In addition, given considerable heterogeneity among depressed individuals to respond to different treatments, future studies should also be aimed at determining whether digital phenotyping may have a role in the prediction of response or non-response thereby allowing for more accurate initial treatment selection or more timely adjustment of treatment to optimize outcome.

## Data Availability Statement

The datasets presented in this article are not readily available because Data include sensitive information. Requests to access the datasets should be directed to ppedrelli@mgh.harvard.edu.

## Ethics Statement

The studies involving human participants were reviewed and approved by MGH and MITIRBs. The patients/participants provided their written informed consent to participate in this study.

## Author Contributions

PP, SF, DI, JA, and RP were responsible for the conceptualization of the study. PP and SF wrote the original draft. SF, AG, and DB performed the formal analysis that was supervised by SF and RP. All the authors reviewed and edited the manuscripts.

## Conflict of Interest

RP served as cofounder and chairman of the board for Empatica, which manufactured the wearable sensors used to collect a subset of the data used in the study. She owned stock in Empatica and served as part-time consultant and chief scientist for them. PP also received royalties from MIT for patents. She was an inventor on related to wearable technology; however, none of these are directly related to this work. DM has received research support from Nordic Naturals and heckel medizintechnik GmbH. He has received honoraria for speaking from the Massachusetts General Hospital Psychiatry Academy, Harvard Blog, and PeerPoint Medical Education Institute, LLC. He also works with the MGH Clinical Trials Network and Institute (CTNI), which has received research funding from multiple pharmaceutical companies and NIMH. The remaining authors declare that the research was conducted in the absence of any commercial or financial relationships that could be construed as a potential conflict of interest.
