# Supplementary material for: Monitoring Changes in Depression Severity Using Wearable and Mobile Sensors
Source: Front Psychiatry. 2020 Dec 18;11:584711. doi: 10.3389/fpsyt.2020.584711 (PMC7775362; doi:10.3389/fpsyt.2020.584711)
Supplement: Supplementary file 1 [file Data_Sheet_1.pdf]

SUPPLEMENT

A.1 Data preprocessing and features extraction

A.2 Model

A.3. Weighted stationary latitude and longitude SD

A.4. Correlations

A.5 Acceptability

A.6. References

Table 1. List of Features by modality

A.1 DATA PREPROCESSING AND FEATURES EXTRACTION

Features derived from the wrist sensors

Electrodermal activity (EDA)

E4 sensors worn on each wrist captured continuous EDA via the measurement of skin conductance (4Hz sampling rate), temperature (4Hz sampling rate), and 3-axis accelerometer data (32 Hz sampling rate). In order to better understand the user's behavior within the day, we introduce 6-hour intervals, labeled as morning, afternoon, evening, and night. The 6-hour interval provides a balance between granularity and ratio of missing values. We also calculate aggregate daily measures. Any feature explained below has been calculated for all these intervals.

We first filtered out the EDA signal when the corresponding skin temperature was below 31°C to exclude the measurements when the sensor was not worn. Then we applied the 6th order Butterworth low-pass filter (1Hz cutoff frequency). We calculated mean EDA and the fraction of time the sensor was recording the signal. We also computed the number of skin conductance response (SCR) peaks and their average amplitude using the method from Gamboa [Gomboa et al. 2008]. Thus, we also encoded asymmetry in EDA between the right and left wrist in different ways: the difference between average EDA value, difference between number of SCRs, and difference between SCL and SCR signals using Convex Optimization Approach [Greco et al. 2016].

Motion

We applied the 5th order Butterworth low-pass filter (10Hz cutoff frequency) to the accelerometer data. We then translated the output into motion features by calculating the vector magnitude, VM of the z-axis acceleration data using the following formula:

$$VM = \sum_{t=0}^{N} VM_t + \left|R_{(z,t)} - M_z\right|$$

where $R_{(z,t)}$ is the raw accelerometer z-axis sample, M_z is the running mean in a 5-second window of the z-axis signal, and N is the number of raw data samples received in one second. Next, we calculated average, median, and standard deviation of motion for the mentioned time intervals as well as the fraction of time in motion. We also kept meta-data such as the fraction of time within the time interval that the data were not missing.

Sleep

We calculated objective sleep based on accelerometer data for 30 second epochs using the ESS method described in [Borazio et al. 2014]. We calculated sleep duration, sleep onset time (time elapsed since noon), maximum duration of uninterrupted sleep, number of wake-ups during the night, and the time of waking up (time elapsed since midnight). We also computed a sleep regularity index (SRI):

$$SRI = \frac{\left[1+\left[\frac{1}{(T-\tau)}\right]\int_0^{T\tau} s(t)s(t+\tau)dt\right]}{2}$$

where data were collected for y = [0, T], τ = 24, s(t) = 1 during sleep and s(t) = −1 during wake. The SRI ranges between 0 (highly irregular sleep) and 1 (consistent sleep every night). We also included meta-data such as the fraction of time that data were being recorded over nighttime (between 8pm-9am) as well as over the period of 24 hours.

Passive features derived from the phone

Social Interaction through the phone

We utilized Movisens [Movisens] on Android to collect measures of how the participant is using his or her mobile phone and how s/he is interacting with other people using the mobile phone. More specifically, we captured meta-data of calls, text messages, app usage, display on/off behavior, and location. Passive data were captured 24/7. The content of the calls/texts, actual phone numbers, websites visited, and the content of the applications were not collected. Following the steps of previous researchers in generating features from passive phone data [Jaques et al. 2015], we introduce 3-hour intervals in order to better understand the user's daytime behavior. For example, [6am-9am] represents early morning while [9pm-12am] corresponds to late evening. We also calculate aggregate daily measures. For quantifying call data, we calculate the number of incoming, outgoing, and missed calls daily and over the 3-hour periods within the day. In a similar manner, we calculate mean, median, and standard deviation (SD) of the duration of incoming, and outgoing calls. Finally, we calculate the incoming/outgoing ratio both for the number of calls and the duration of calls on a daily basis. For quantifying SMS data, we use a similar approach, we calculate the number of incoming and outgoing texts daily and over 3-hour periods within the day. We also calculate a daily incoming/outgoing ratio of the number of text messages received or sent respectively.

Phone Engagement

Given that turning the display on/off is also an indication of phone usage, we examined the mean, median, and SD of duration of screen on within the mentioned intervals. We also calculate the number of the times the screen has been turned on over these periods. Note that these two correspond to different behaviors; Long screen-on duration is related to actively using the phone while a great number of screen-ons is related to consistently checking the phone which might be a sign of anxiety or anticipation.

Location

For location data, we calculate mean, median, and SD of latitude and longitude along with the number of data points that have been captured for each time period. We calculate total location mean, median, and SD by averaging values from latitude and longitude.

Weighted stationary latitude and longitude SD

Higher level features were defined based on location data. To capture the variation of the locations the participant visited during the day 'weighted stationary latitude and longitude SD' was computed as follow:

1: Finding stationary datapoints: For each recorded location datapoint, instantaneous speed of the participant was calculated. Precisely, the speed was calculated by dividing the distance from the last location datapoint in meters by the time difference between the current and previous location

recording in seconds. Any point with the instantaneous speed smaller than 0.3 m/s was considered “stationary”. 2: Calculating weighted standard deviation of the stationary points: Considering all the stationary data points, the time difference between every consecutive pairs was calculated. Note that the time difference between stationary datapoints is not uniformly distributed. For example, a participant may have spent a different amount of time in their office or at home or in the bus station waiting for the bus. To calculate standard deviation by considering the time spent at each location, sqrt(cov(latitude, time_diff) was calculated and similarly for longitude.

App usage

To quantify app usage, we first encoded the app category using the following list: game, email, web, calendar, communication, social, maps, video streaming, photo, shopping, and clock and then, we calculated the total duration and the number of app category usage in the different mentioned time intervals.

The extracted features from all modalities are listed in Table 1.

Features transformation and selection

Combining the carefully-crafted features resulted in 877 features for our dataset. Compared to the small number of data points we have, this number of features can easily result in over-fitting the model to the training set, so we took additional steps to prevent that. One possibility is to use regularization tricks such as L1 to enforce selection of only a small number of features. However, for features that are non-linearly related, transforming the features into a new space through a non-linear transformation can be more beneficial. For example, several noisy measurements of a similar phenomenon may not be informative on their own, but a transformed version of them can be a better predictor. Toward this end, we tested PCA, kernel PCA with radial-basis function kernel, and truncated SVD methods to reduce the dimensionality of our feature set. We ended up using the kernel PCA method as it returned the best results. We bound the number of selected features while keeping as few features as possible to explain the variance of data.

A.2 MODEL

We used python to develop all our code. In particular we used scikit-learn library (https://scikit-learn.org/stable/) for machine learning programming. In particular, for the Random Forest method we used the following library: https://scikit-learn.org/stable/modules/generated/sklearn.ensemble.RandomForestRegressor.html

For the Boosting method, we used the following library:https://scikit-learn.org/stable/modules/generated/sklearn.ensemble.AdaBoostRegressor.html

Our code is available online at this address:

https://github.com/MITMediaLabAffectiveComputing/MIT_MGH_GrandChallengeStudy

We used a customized ensemble method combining the results of different regressors to get a more robust model.

Regression methods

We used several regressors to estimate intermediate HDRS score. The regression methods include lasso, ridge, and elasticNet which use L1, L2, and a combination of the two as regularization metrics, respectively. In addition, to be robust against outliers or errors in formulation of the model, we include Theil-Sen estimator, random sample consensus (RANSAC), and huber algorithms. These models have a built-in sampling procedure that allows a fraction of data points to be outliers.

Ensemble method

Finally, we combine the results from these different regressors to get a more robust estimator. The ensemble method first finds a set of k nearest neighbors from the training set for each point. It then chooses the model that performs best on that set as the estimator for this point. The heuristic behind this method is that slight modifications in the feature set do not change the output drastically. Thus, if a classifier is working well on similar points, chances are it works well for the current point, as well. Looking at k nearest points as opposed to only the most similar point is for smoothing purposes. Note that as the points become higher dimensional, the distance between them becomes less meaningful in explaining similarity between the points. Thus, we only use the first 5 reduced features based on kernel-PCA and create a KD tree and find the k nearest neighbors to the point at hand.

Model training and testing

To prevent model overfitting we used a standard procedure. Specifically, we run 10-fold cross-validation. Its purpose is to assess how the results of a statistical analysis will generalize to an independent data set. We split the data set into 10 random subsamples and during 10 rounds, 9 subsamples form the training set whereas the remaining subsample is used for validation. At the end, the performance results of the 10 rounds are averaged to produce a single estimation which is a standard approach.

We run the cross-validation 5 times for every model and split scenario using a different random initialization seed. We used the following seed values: 12, 76, 3465, 12345 and 14523. The reported performance is the average of the results obtained at each run.

The data were divided in training test and three scenarios, time-split, user-split and random split. In the paper we described the method and results of the time split and user split.

Random testing scenario

In addition to evaluating the model using the user-split and time-split scenarios we also examined a random-testing scenario. In the random-split scenario, data were split randomly into 80% train set and 20% test set. Although this is a common evaluation methodology, it is not informative in this setting. This scenario includes a simplifying assumption that the datapoints are independent and identically distributed (i.i.d.). However, the presence of multiple patients contributing to the dataset and of a chronological order contradict the i.i.d. assumption. Given that the split is agnostic to the patient and the chronological order of the data points, data from future visits of a patient may be included in the training set and previous visits of the same patient may be assigned to the test set. We include this scenario due to its common usage.

In the random-split scenario, MAE was 3.49±0.49, in the model including features from the mobile, 3.84±0.49 in the model including features from the wearables and 3.75±0.57 in the model with all the features. Values for RMSE in the model including features from the mobile were 4.68±0.57, in the model including features from the wearables was 4.81±0.44 in the model with all the features was 4,89±0.53. The clinician-rated HDRS scores and the estimated HDRS scores had 0.51 correlation coefficient.

A.3. Correlations

We calculated the Pearson correlation coefficient between the clinician-rated HDRS scores and the estimated HDRS scores on the hold-out test set in each training scenario. In the user-split and random-split scenarios we used 20% of the data points (=.2 * 155 = 31) for calculation of the

correlations. In the time-split scenario, we used 2 data points from each user for the calculation of the correlations and therefore we had 2*31 = 62 data points.

A.4 Acceptability

We evaluated the impact of technical problems on the E4 wristbands acceptability. After we removed the days with technical problems, the adherence increased from 77% to 94% and from 70% to 90% for the left and right-hand devices accordingly. Technical problems usually led to periods of three or more consecutive days when the data was not uploaded from the participant's sensor. The team confirmed these periods of technical problems by reviewing the study notes. Figures 1 and 2 show the average number of hours the E4 was worn by of each user computed over the total days they were in the study and over the days without technical problems.

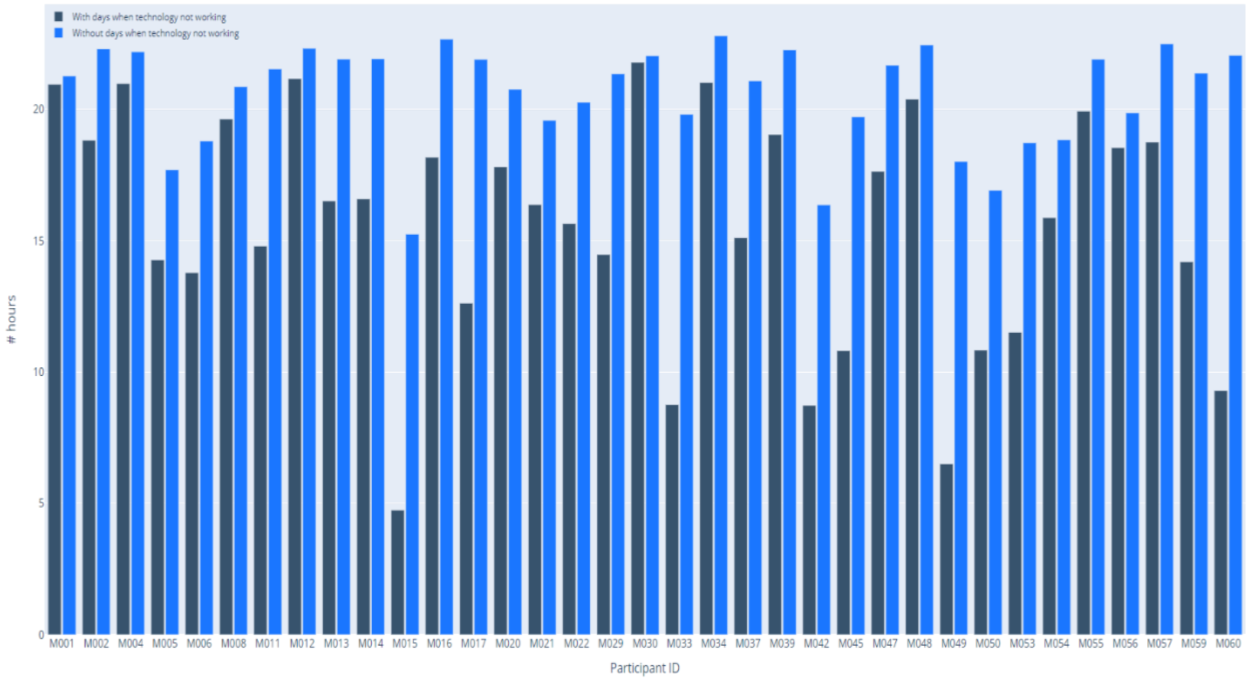

Figure 1: Number of hours E4 wearables was worn on the left hand by each participant overall and during the days without technical problems

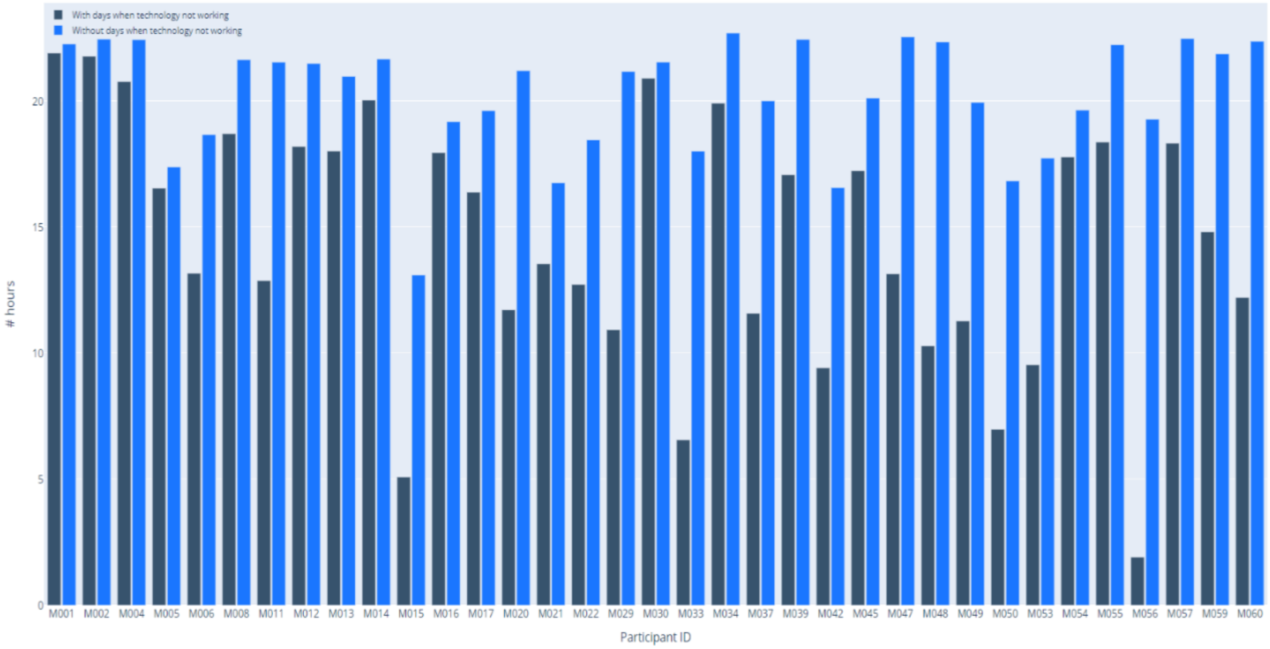

Figure 2: Number of hours E4 wearables was worn on the right hand by each participant overall and during the days without technical problems

A.6. References

Borazio, M., Berlin, E., Kucukyildiz, N., Scholl, P., &. Van Laerhoven, K. “Towards benchmarked sleep detection with wrist-worn sensing units,” in Healthcare Informatics (ICHI), 2014 IEEE International Conference on. IEEE, 2014, pp. 125–134.

Camm, A. J., Malik, M., Bigger, J. T., Breithardt, G., Cerutti, S., Cohen, R. J., ... & Lombardi, F. (1996). Heart rate variability: standards of measurement, physiological interpretation and clinical use. Task Force of the European Society of Cardiology and the North American Society of Pacing and Electrophysiology.

Gamboa, H. (2008). Multi-modal behavioral biometrics based on HCI and electrophysiology. *PhD ThesisUniversidade*.

Ghandeharioun, A., Fedor, S., Sangermano, L., Ionescu, D., Alpert, J., Dale, C., ... & Picard, R. (2017, October). Objective assessment of depressive symptoms with machine learning and wearable sensors data. In *2017 Seventh International Conference on Affective Computing and Intelligent Interaction (ACII)* (pp. 325-332). IEEE.

Greco A., Valenza G., Lanata A., X Scilingo V, and Citi, L. “cvxeda: A convex optimization approach to electrodermal activity processing,” IEEE Transactions on Biomedical Engineering, vol. 63, no. 4, pp. 797–804, 2016.
Jaques, N., Taylor, S., Azaria, A., Ghandeharioun, A., Sano, A., & Picard, R. (2015, September). Predicting students' happiness from physiology, phone, mobility, and behavioral data. In *2015*

International Conference on Affective Computing and Intelligent Interaction (ACII) (pp. 222-228). IEEE.

MovisensXS, "eXperience Sampling for Android," https://xs.movisens.com, 2012, online; accessed January 2020.

Table1

List of features by modality

Device	Modality	Feature	Aggregation	Number of Features	Comments
E4	EDA	EDA SCL	Left, right wrist and difference between left and right, processed for motionless and with-motion periods, normalized and non-normalized, using standard method and convex optimization approach averaged over 24hrs, night (midnight – 6am), morning (6am-noon), afternoon (noon-6pm), evening (6pm-midnight)	3 x 2 x 2 x 2 x 5 = 120	More details about the EDA features processing are provided in Ghandeharioun et al.2017
		EDA SCR	Left, right wrist and difference between left and right, processed for motionless and with-motion periods, normalized and non-normalized, number of peaks and amplitude averaged over 24hrs, night (midnight – 6am), morning (6am-noon), afternoon (noon-6pm), evening (6pm-midnight)	3 x 2 x 2 x 2 x 5 = 120	

	Motion	Motion magnitude	Left and right wrist mean, median, STD over 24hrs, night (midnight – 6am), morning (6am-noon), afternoon (noon-6pm), evening (6pm-midnight)	2 x3 x 5 =30	The formula for the Motion magnitude is provided in Ghandeharioun et al. 2017
		Time in motion	Fraction of time when the person is in motion calculated over 24hrs, night (midnight – 6am), morning (6am-noon), afternoon (noon-6pm), evening (6pm-midnight)	5	The motion detection is based on the thresholding of the accelerometer vector magnitude as described in Ghandeharioun et al. 2017
	Sleep	Time in sleep	Sleep time over 24hrs and night	2	
		Sleep characteristics	Sleep onset time, maximal night uninterrupted sleep, number of wakeups during night, wakeup time, sleep regularity index	5	Sleep Regularity Index formula provided in Ghandeharioun et al. 2017
	HR/HRV	HRV time domain	Standard HRV metrics (AVNN, pNN50, rMSSD, SDANN, sDNNIDX, rrSDNN) averaged or STD over 24hrs, night (midnight – 6am), morning (6am-noon), afternoon (noon-6pm), evening (6pm-midnight)	6 x 2 x 5 = 60	Details on HRV variables calculation provided in Camm et al. 1996
		HRV frequency domain	PSD of the high-frequency, low-frequency and very low-frequency, HF/LF ratio, total HRV frequency signal averaged, STD over 24hrs, night (midnight – 6am), morning (6am-noon),	5 x 2 x 5 = 50	

			afternoon (noon-6pm), evening (6pm-midnight)		
		HR	Averaged and STD heart rate over 24hrs, night (midnight – 6am), morning (6am-noon), afternoon (noon-6pm), evening (6pm-midnight)	2 X 6 = 12	
Phone	Location	Latitude, longitude, % of time at home, total distance, transition time, stationary time	Mean, median and STD over 24hrs, night (midnight – 6am), morning (6am-noon), afternoon (noon-6pm), evening (6pm-midnight)	6 x 3 x 5 = 90	
	App usage	Use of the calendar, clock, communication, email, Facebook, game, maps, photo, shopping, web, YouTube apps	Use time and use count over 24hrs, night (midnight – 6am), morning (6am-noon), afternoon (noon-6pm), evening (6pm-midnight)	11 x 2 x 5 =110	
	Phone call	Incoming calls dismissed missed, responded, outgoing calls not reached, responded	Count; duration average, median, STD, sum over 24hrs, night (midnight – 6am), morning (6am-noon), afternoon (noon-6pm), evening (6pm-midnight)	5 X 6 X 5 = 150	
	SMS	Incoming SMS, outgoing SMS	Count, length, average, median, STD, sum over 24hrs, night (midnight – 6am), morning (6am-noon), afternoon (noon-6pm), evening (6pm-midnight), day hours (8am-10pm)	2 X 6 X 6 = 72	
	Display	On duration	Average, median, sum, count, STD over 24hrs, night (midnight – 6am), morning (6am-noon), afternoon (noon-6pm), evening (6pm-midnight), day hours (8am-10pm)	5 X 6 = 30	

Online	Weather	Apparent temperature	Max, min, STD, average	4	The weather variables are provided by an online service Dark Sky. The documentation of the variables is provided at https://darksky.net/dev/docs#response-format
		Cloud cover, Humidity, Precipitation probability, pressure, wind speed	Average, STD	5 X 2 = 10	
		Dew point, Moon phase, UV index, visibility	Average	4	
		Insolation time	Sum	1	
		Precipitation intensity	Max, average,	2	
